# The assessment and validation of the depression, anxiety, and stress scale (DASS-21) among frontline doctors in Pakistan during fifth wave of COVID-19

**DOI:** 10.3389/fpubh.2023.1192733

**Published:** 2023-06-16

**Authors:** Muhammad Umar Nadeem, Steve J. Kulich, Ijaz Hussain Bokhari

**Affiliations:** ^1^SISU Intercultural Institute (SII), Shanghai International Studies University (SISU), Shanghai, China; ^2^School of Commerce and Accountancy, University of Management and Technology (UMT), Lahore, Punjab, Pakistan

**Keywords:** COVID-19, depression, anxiety, stress, doctors, Pakistan

## Abstract

**Objective:**

The study aims to document sociodemographic features, address the symptoms and levels of depression, anxiety, and stress among frontline doctors in Pakistan, and validate the depression, anxiety, stress scale (DASS-21) on the context of Pakistan.

**Method:**

A cross-sectional survey was conducted throughout the regions of Pakistan on frontline doctors to document their sociodemographic patterns and the levels of depression, anxiety, and stress while dealing with the fifth wave (Omicron-variant) of the coronavirus (SARS-CoV-2) pandemic in Pakistan (December 2021–April 2022). Respondents (*N* = 319) were recruited through a snowball sampling process.

**Results:**

Though previous literature reported declines in psychological symptoms after earlier waves of COVID-19, these DASS-21 findings show that as the pandemic has worn on, frontline doctors in Pakistan are having considerable personal symptoms of depression (72.7%), anxiety (70.2%), and stress (58.3%). Though specifically related to the COVID-19 pandemic, they rated only moderate levels of depression and stress, however they reported severe levels of anxiety. The results also revealed a positive correlation between depression and anxiety (*r* = 0.696, *p* < 0.001), depression and stress (*r* = 0.761, *p* < 0.001), and anxiety and stress (*r* = 0.720, *p* < 0.001).

**Conclusion:**

Through the application of all required statistical procedures, DASS-21 is validated in the cultural context of Pakistan among this group of frontline doctors. The findings of this study can provide new directions for the policy makers (government and hospitals' administration) of Pakistan to focus on the mental wellbeing of the doctors under similar enduring public health crises and to protect them from short- or long-term disorders.

## 1. Introduction

The coronavirus (SARS-CoV-2) was reported to have emerged from the city of Wuhan in Hubei province in China and later reported to spread rapidly in different parts of the world within the following months. Its most common symptoms have been equated with flu, high fever, dry cough, sore throat, loss of taste, with frequent impact on the lungs and respiratory system creating breathing difficulties, and with some cases leading to death (1, 2). The speedy transmission, heavy upsurge of infections, and associated deaths have created a sense of panic across the world (3). As the pandemic wave(s) spread, most countries declared the novel corona virus to be a public health emergency and took precautionary measures such as social distancing, isolation, quarantines, and wearing masks (4) to reduce its dispersion (5) aiming to protect their citizens.

Other than physical health, implementation of lockdowns (restricting populations to stay in and work from home) along with inadequate information or uncertain measures to protect themselves or vulnerable loved ones have created varying types of psychological distress among people worldwide (6). Fears of being infected or isolated in quarantines have adversely affected many as they felt compelled to distance themselves from their peers, colleagues, families, and other social contacts (7). Seeking to deal with the uncertainties and possible implications of COVID-19 has impacted human psychology at many levels, including increasing fear, stress, anger (8), depression, anxiety, and in worst case scenarios, suicidal tendencies (9). It has also been reported that pandemic distress coupled with certain extreme preventive measures could trigger other pre-existing mental health diseases and even induce novel symptoms in those who previously did not have any mental health issues (10).

COVID-19 pandemic related studies across various regions have documented different psychological factors that directly and indirectly affect the mental health of almost every segment of the population (11). Special attention has been given to the impacts on medical workers around the world (12–15) as they were both directly exposed to COVID-19 patients and psychologically faced with additional self- and other-care challenges. Studies conducted in diverse cultural contexts such as, Israel, Turkey, Egypt, India, the United States, Saudi Arabia, China, Kuwait, Iran, Poland, and Bangladesh (16–26) have noted and examined the unique issues and implications facing medical staff during the COVID-19 pandemic. However, neither the context nor challenges facing doctors in Pakistan have been widely reported in recent literature especially during the fifth wave of COVID-19.

Pakistan presents an interesting and important case for a national context as a highly populated (220 million) under-developed country and one already facing the multiple challenges of an energy crisis, weak economy, and political instability. A health emergency was declared in Pakistan right after the initial infected COVID-19 cases gained momentum. Partial and smart lockdowns, vaccinations, and improved treatment policies have helped Pakistan to control the transmission of the virus to protect the general citizens of the country (27).

An earlier investigation about the COVID-19 pandemic on seven different Asian countries (including Pakistan) found that the people of Thailand and Pakistan scored very high on the depression anxiety stress scale (DASS-21) as compared to the other countries (28). A study on the Punjab province of Pakistan reported that 21.9% of depression and 21.4% of anxiety symptoms were witnessed among the health care workers (HCW) and that the most affected population was medical doctors (29). It is also found that 79.7% of the HCW in Pakistan were having very high levels of and severe anxiety issues concerning COVID-19 (7). Researchers interviewed thirteen young doctors and found that they were experiencing psychological distress in the form of increased stress, fear, and anxiety after the emergence of the COVID-19 pandemic in Pakistan (30). Another sample reported that 43% of anxiety/depression prevailed among the frontline doctors of Pakistan in 2020 (31). Clearly, HCW in Pakistan have been more exposed to COVID-19 as elsewhere, and as an important health service sector that every country looks to in such distressing pandemic situations, their responses, wellbeing, and mental health cannot be overlooked.

The abovementioned studies provide evidence that HCW and frontline doctors are among the most vulnerable populations at a higher risk who are also more exposed to COVID-19 cases on a frequent and long-term basis as compared to ordinary people (in Pakistan as in other nations). Unfortunately, most of previous studies were completed during the initial waves of COVID-19 and did not adopt a well-established scale (like the DASS-21) to document the levels of depression, anxiety, and stress specifically of doctors. DASS-21 is widely considered to effectively address the symptoms of depression, anxiety, and stress (32) which indicate the mental health of the general populace rather than a clinical population. Research gaps exist in not examining the fifth wave of COVID-19, application of DASS-21, and specifically not yet targeting the frontline doctors in Pakistan. Therefore, the current study aims to address and consider the connections between these previously unexplored areas by incorporating the DASS-21 to apply this instrument to the frontline doctors of Pakistan during fifth wave of COVID-19.

## 2. Methods

### 2.1. Respondent and procedures

An online survey (through Google Forms) was created to capture the responses of frontline doctors who were directly dealing with the COVID-19 patients during the fifth wave in Pakistan. In the scenario of this emergency, limited access due to pandemic measures, and seeking broader reach, we resorted to utilizing a snowball sampling technique and approached a couple of doctors that could be accessed to participate in this study and help recruit others. The survey form was shared with them through different social networking platforms. The consent form clearly stated at the top of the survey that their responses and identities would be kept confidential, and if they feel uncomfortable while filling in the survey that they can leave it at any stage. The overall process of data collection took 3 months: starting from February 2022 and ending in April 2022 (at the time that the fifth was considered over).

Considerations regarding the selected sample size include: First, the recommended, calculated minimum sample size was 10 participants for each scale item. Regardless of the number of items on a scale, at least 210–310 participants are recommended for factor analysis (33). The sample size (*N* = 319) in our study was sufficient according to the ideal ratio of items (10:1). Second, the former relevant studies have considered healthcare workers or professionals (including doctors) as their samples (29–31). Representative size may be questioned, yet the current study has only focused on the frontline doctors compared to the entire medical staff (representation is intentionally limed to this select and important group). Third is the size needed for analysis, whereby structural equation modeling (SEM) requires a minimum of 200 and a maximum of 500 samples for the data analysis regarding the estimation of good results (34). Therefore, the samples (*N* = 319) of this current research were in between these two thresholds and considered adequately suited for final analysis.

### 2.2. Survey instrument

The survey form was entirely designed in English with two major sections. The first section elicited standard sociodemographic features such as area, gender, age, etc. and for section two, the DASS-21 instrument (35) was adopted to measure the levels of depression, anxiety, and stress of the doctors. Section one was further classified into ten major sociodemographic questions including area, gender, age, marital status, workplace, job title, current area of practice and work. In addition, two questions about the media preference and the consumption of COVID-19 related news on that specific media channel/portal were also included in this section. To assess psychological states, the DASS-21 instrument contains a total of 21 items with 7 items for each of the three dimensions (depression, anxiety, and stress) respectively. Participating medical doctors were encouraged to rate their responses about the current situation which they were facing in the midst of the fifth wave of COVID-19. A four-point Likert type scale was incorporated to capture their responses ranging from 0 (did not apply to me at all) to 3 (applied to me very much) to avoid mid-point non-meaningful responses. The lower scores represent a normal range; however, the higher scores indicate a more severe emotional situation affecting the doctors. This instrument has previously been shown to exhibit very high reliability and validity and used in a very recent study (32). Furthermore, the validity and reliability of DASS-21 during the COVID-19 period have also been confirmed (36). It has robust validity and reliability values. We therefore employed the original and still widely used version of the scale for the current study.

### 2.3. Statistical analysis

All statistical procedures were first evaluated using the Statistical Package for Social Sciences (SPSS) version 23.0 and later the reliability and validity of DASS-21 were processed by the Analysis of a Moment Structures (AMOS) version 23.0. In SPSS, the descriptive statistics and one-way ANOVA were performed to report the frequency and percentages of all sociodemographic features of the participants and to document the significant differences among each demographic sub-section. In addition, the individual scores for depression, anxiety, and stress as well as the overall scores of DASS-21 were also evaluated. All essential aspects for testing the reliability and validity of DASS-21 were examined in AMOS.

## 3. Results

### 3.1. Respondents' features

In this study, a total of 319 frontline doctors completed the survey form. The sociodemographic details of the respondents are presented in Table 1 (through SPSS). Many frontline doctors belonged to the Pakistan region of Punjab (*N* = 155, 48.6%) and 87.8% of the respondents were young with an age range between 20 and 30. Female doctors heavily dominated the sample set with 73%. Regarding marital status, those separated/divorced participants were minimal (only 1.2% of the sample) and most were single (*N* = 227, 71.2%). In terms of their workplace and job title, 87.8% of the doctors were directly associated with the hospitals and 60.5% were titled as the house officers. Most of them were working in the private sector (*N* = 176, 55.2%) and performing their duties in wards (*N* = 143, 44.8%). Regarding media use, a large majority of doctors (*N* = 280) preferred digital media over newspapers and television. 63% of the frontline doctors reported they consume any form of media for less than an hour daily, mainly to update themselves on news regarding COVID-19, thus can be considered minimal and functional or information-oriented media consumers.

**Table 1:** Features of respondents and one-way ANOVA results.

**Variables**		***N* (%)**	**Depression**		**Anxiety**		**Stress**	
			**F**	***p***	**F**	***p***	**F**	***p***
Area	Punjab	155 (48.6)	0.513	0.798	0.286	0.943	0.333	0.919
	Sindh	25 (7.8)						
	KPK	24 (7.5)						
	Baluchistan	9 (2.8)						
	AJK	13 (4.1)						
	Gilgit-Baltistan	2 (0.6)						
	Islamabad (ICT)	91 (28.5)						
Gender	Male	86 (27.0)	12.542	< 0.001	5.505	0.020	6.470	0.011
	Female	233 (73.0)						
Age	20–30	280 (87.8)	2.488	0.061	3.368	0.019	2.825	0.039
	31–41	18 (5.6)						
	42–52	13 (4.1)						
	53–63	8 (2.5)						
Marital Status	Single	227 (71.2)	3.571	0.014	6.283	< 0.001	4.375	0.005
	Married	88 (27.6)						
	Separated	1 (0.3)						
	Divorced	3 (0.9)						
Workplace	Hospital	280 (87.8)	0.139	0.936	2.205	0.087	2.639	0.050
	Health clinic	28 (8.8)						
	District health office	8 (2.5)						
	State health office	3 (0.9)						
Job Title	House officer	193 (60.5)	5.069	0.007	7.110	0.001	6.390	0.002
	Medical officer	84 (26.3)						
	Specialist	42 (13.2)						
Current area of practice	Public sector	132 (41.4)	1.255	0.263	1.200	0.274	2.299	0.130
	Private sector	187 (58.6)						
Current area of work	Emergency	55 (17.2)	0.520	0.595	0.087	0.917	0.798	0.451
	OPD	121 (37.9)						
	Ward	143 (44.8)						
What is your media preference?	Digital media	280 (87.8)	0.090	0.914	0.831	0.437	0.170	0.844
	Television	35 (11.0)						
	Newspaper	4 (1.3)						
News about COVID-19 (per day)?	< 1 h	201 (63.0)	2.588	0.053	8.058	< 0.001	3.185	0.024
	1–2 h	76 (23.8)						
	3–4 h	24 (7.5)						
	More than 4 h	18 (5.6)						

### 3.2. Levels of depression, anxiety, and stress of frontline doctors

The significant differences regarding the three facets (depression, anxiety, and stress) of DASS-21 among each demographic feature were evaluated based on the one-way ANOVA results. The findings revealed that there were significant differences between the sub-categories of gender (*F* = 12.542, *p* < 0.05; *F* = 5.505, *p* < 0.05; *F* = 6.470, *p* < 0.05), marital status (*F* = 3.571, *p* < 0.05; *F* = 6.283, *p* < 0.05; *F* = 4.375, *p* < 0.05), and job title (*F* = 5.069, *p* < 0.05; *F* = 7.110 *p* < 0.05; *F* = 6.390, *p* < 0.05) of the frontline doctors in reporting depression, anxiety, and stress. In addition, significant differences were also witnessed among the age (*F* = 3.368, *p* < 0.05; *F* = 2.825, *p* < 0.05) and daily media coverage consumption (*F* = 8.058, *p* < 0.05; *F* = 3.185, *p* < 0.05) between the doctors concerning the levels of anxiety and stress. However, the other sub-categories of respondents' profile (i.e., area, workplace, area of practice and others) did not have any statistically significant differences regarding the levels of depression, anxiety, and stress. To highlight these findings, the significant values obtained from one-way ANOVA for depression, anxiety, and stress against every sociodemographic feature are stated in Table 1.

The overall trend (Table 2) of the scores revealed that the frontline doctors were having noticeable symptoms of all three: depression (*N* = 232, 72.7%), anxiety (*N* = 224, 70.2%), and stress (*N* = 186, 58.3%). The depression symptoms range among respondents were extremely severe (22.9%), severe (10.7%), moderate (23.8%), and mild (15.4%) respectively. Less intense, the symptoms of anxiety ranged from 8.5% as mild, 14.4% as moderate, 11.6% as severe, and 35.7% as extremely severe in frontline doctors. The participants' reported stress symptoms ranging from 16.3% extremely severe, 17.6% severe, 14.7% moderate, and 9.7% mild. Furthermore, the mean scores for DASS-21 (*M* = 51.69) and its subscales were also calculated to evaluate the exact level of depression (*M* = 17.31), anxiety (*M* = 15.24), and stress (*M* = 19.14) among the frontline doctors. The mean scores highlighted that the frontline doctors of Pakistan were having severe levels of anxiety and moderate levels of depression and stress.

**Table 2:** Levels of depression, anxiety, and stress.

	**Depression**			**Anxiety**			**Stress**		
	**Limit**	***N***	**%**	**Limit**	***N***	**%**	**Limit**	***N***	**%**
Normal	0–9	87	27.3	0-7	95	29.8	0–14	133	41.7
Mild	10–13	49	15.4	8-9	27	8.5	15–18	31	9.7
Moderate	14–20	76	23.8	10-14	46	14.4	19–25	47	14.7
Severe	21–27	34	10.7	15-19	37	11.6	26–33	56	17.6
Extremely severe	28+	73	22.9	20+	114	35.7	34+	52	16.3

### 3.3. Reliability and validity of the DASS-21

An assessment of DASS-21 was carried out in AMOS, to reconfirm its reliability and validity among the frontline doctors in the context of Pakistan. The process of evaluation was done by considering different approaches such as alpha values, composite reliability (C.R.), confirmatory factor analysis (CFA), construct and convergent validity, and fitness indices in AMOS. The model has fulfilled the minimum required values suggested by the literature (**34**) regarding CFA (>0.50) and reliability (Table 3). The DASS-21 showed an overall excellent internal consistency reliability (Cronbach's *α* = 0.953, McDonald's *ω* = 0.954) as well as for its sub-scales such as, Depression (Cronbach's *α* = 0.913, McDonald's *ω* = 0.917), Anxiety (Cronbach's *α* = 0.883, McDonald's *ω* = 0.884), and Stress (Cronbach's *α* = 0.928, McDonald's *ω* = 0.928). Furthermore, for each factor, all the square roots of average variance extracted (AVE) are highlighted in bold and shown (Table 4) to be greater than the coefficients or off-diagonal elements in the corresponding rows and columns, thus establishing evidence of discriminant validity (>0.70).

**Table 3:** DASS-21 items and loading.

**DASS-21 items**	**Depression**	**Anxiety**	**Stress**
DN1-I could not seem to experience any positive feeling at all	0.730		
DN2-I found it difficult to work up the initiative to do things	0.716		
DN3-I felt that I had nothing to look forward to	0.789		
DN4-I felt downhearted and blue	0.865		
DN5-I was unable to become enthusiastic about anything	0.850		
DN6-I felt I was not worth much as a person	0.751		
DN7-I felt that life was meaningless	0.743		
AT1-I was aware of dryness of my mouth		0.510	
AT2-I experienced breathing difficulty		0.621	
AT3-I experienced trembling		0.713	
AT4-I was worried about situations in which I might panic and make a fool of myself		0.746	
AT5-I felt I was close to panic		0.800	
AT6-I was aware of the action of my heart in the absence of physical exertion		0.772	
AT7-I felt scared without any good reason		0.827	
ST1-I found it hard to wind down			0.762
ST2-I tended to over-react to situations			0.808
ST3-I felt that I was using a lot of nervous energy			0.853
ST4-I found myself getting agitated			0.835
ST5-I found it difficult to relax			0.801
ST6-I was intolerant of anything that kept me from getting on with what I was doing			0.811
ST7-I felt that I was rather touchy			0.757

**Table 4:** Results of validity and reliability.

	***α***	**C.R**.	**AVE**	**Depression**	**Anxiety**	**Stress**
Depression	0.913	0.915	0.608	**0.780**		
Anxiety	0.883	0.881	0.519	0.696^***^	**0.825**	
Stress	0.928	0.929	0.647	0.761^***^	0.720^***^	**0.805**

*** ρ < 0.001. Bold values represent the establishing evidence of discriminant validity (>0.70).

**Figure 1:**
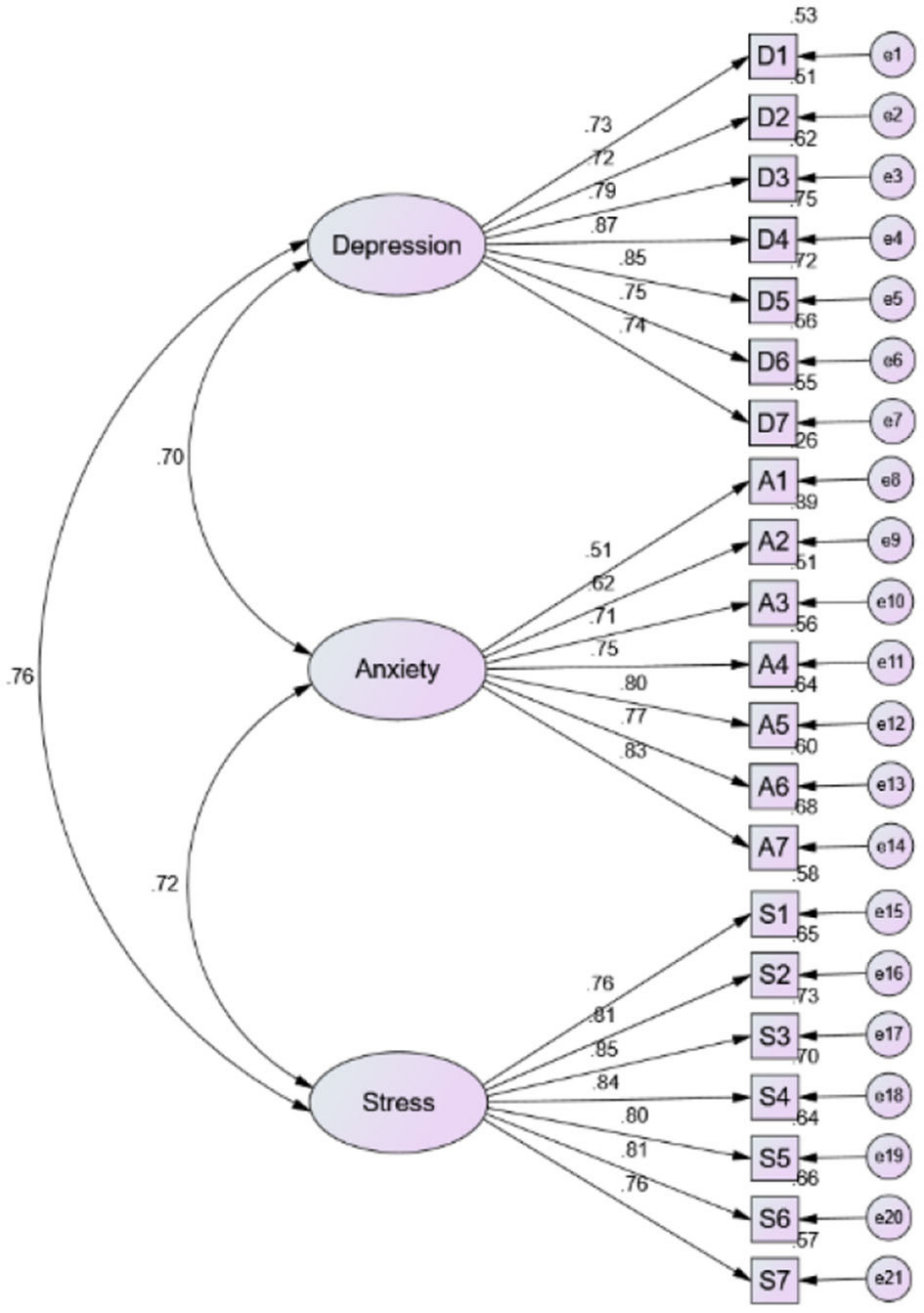
DASS-21.

Table 4 also indicates that the C.R. values for depression (0.915), anxiety (0.881), and stress (0.926) were relatively higher than the minimum limit of acceptance (>0.70). In addition, the values of AVE for depression, anxiety, and stress were 0.608, 0.519, and 0.617 respectively. It reconfirms that the values have crossed the required minimum threshold (>0.50). Lastly, the fitness indices confirmed that the data were well fitted with the measurement model of DASS-21 which indicates the attainment of construct validity: χ^2^ = 302.015, χ^2^/d*F* = 1.67, SRMR = 0.040, GFI = 0.920, NFI = 0.937, IFI =0.973, TLI 0.969, CFI = 0.973, PNFI 0.803, and RMSEA = 0.046 (37, 38). The measurement model of DASS-21 is presented in Figure 1. Therefore, these procedures of instrument testing confirmed that the use of DASS-21 is validated among the frontline doctors in the cultural context of Pakistan.

### 3.4. Correlations

The correlations between depression, anxiety, and stress were also examined in the current study (Table 4). The findings revealed that depression was positively and significantly correlated with anxiety (*r* = 0.696, *p* < 0.001) and stress (*r* = 0.761, *p* < 0.001). Furthermore, a positive and significant association between anxiety and stress (*r* = 0.720, *p* < 0.001) was also witnessed from the findings of this present study.

## 4. Discussion

The study aimed to fill noted gaps to extend pandemic research to document the symptoms and levels of depression, anxiety, and stress among the frontline doctors in Pakistan during the fifth wave of COVID-19, and to validate DASS-21 in the context of Pakistan. Past research in Pakistan tended to focus mainly on HCW but could not be extrapolated to project the special case situation or psychological orientations faced by frontline doctors who had to actively deal with a pandemic that lasted several years and face the unabating needs of COVID-19 patients. Most of the previous research had been carried out during the initial waves of COVID-19 and though contributing much toward general understandings of the psychological effects of a pandemic, had not yet found ways to study the ongoing mental health of frontline doctors, especially during this late stage, in the fifth wave of COVID-19. Most importantly, a well-established scale (DASS-21) had not been previously considered or validated, either in Pakistan or tested for its effectiveness in determining which psychological symptoms arise more prominently in medical or para-medical staff. Therefore, connecting all the above-mentioned limitations, the current study is designed to fill the gaps in the existing literature.

It is evident in this study that 72.7% of doctors were having the symptoms of depression, 70.2% were having anxiety, and 58.3% were dealing with stress arising from the COVID-19 pandemic. The findings revealed that the psychological symptoms reported during the fifth wave of COVID-19 are much higher than the previously documented symptoms had been during the start of pandemic (29). This may be a logical finding from an ongoing pandemic, but has not been studied or confirmed previously, nor the impact expected to this hight degree. In addition, doctors rated moderate levels of depression and stress, but severe levels of anxiety specifically related to COVID-19 issues. The levels are in line with the findings of previous research which reported the severity of anxiety among the HCW in Pakistan (7). It has been reported that the symptoms and levels of depression, anxiety, and stress are more intense over time as compared to the earlier studies (30, 31). In comparison to the normal populace, it seems that mental health of the frontline doctors is seriously affected and considerably worse since the emergence of COVID-19 pandemic, suggesting that their needs may need to be recognized and better dealt with. Even though the treatment system has been improved and multiple vaccines are available and have been administered broadly, medical doctors are still facing psychological challenges.

In terms of the validation of DASS-21, the results of multiple statistical procedures essential for the attainment of reliability and validity of any measurement tool were fulfilled in the current study. There is abundant evidence available in the literature that has confirmed that DASS-21 is a reliable and valid scale for the assessment of depression, anxiety, and stress symptoms among various cultural contexts (32, 35). However, very few studies are available that confirm DASS-21 as a valid measurement tool in the cultural context of Pakistan, and if so, most have been applied to the general public (28) not specifically to the frontline doctors of Pakistan. The findings of this investigation revealed that DASS-21 is a valid and reliable measurement tool to document the depression, anxiety, and stress symptoms of frontline doctors during the fifth wave of COVID-19 on the cultural setting of Pakistan.

Though it has been established that mental health challenges are not limited to ordinary citizens or persons that have already been diagnosed as having mental health diseases, this study shows that those professionals that society relies on most during times of international health crises suffer at higher rates than might be expected. COVID-19 studies have already warned the world that the pandemic will likely have lasting impacts on the masses. The findings of the existing study have reconfirmed their predictions. Continuing in the line of studies that have examined HCW and the medical profession, this study shows even more clearly that even doctors, who are trained to deal with crises and have many such experiences, are not unaffected by its impacts, and in fact, perhaps suffer far more than expected. Pakistan has excellent medical doctor training, and its physicians are expected to perform an important role in the stability of society. Therefore, findings like these on the existence of serious levels of depression, anxiety, and stress among them even (or especially after dealing with several years of this pandemic) cannot be neglected due to the potential adverse effects on the society.

The pandemic may now have subsided, but more research is needed to determine if there are any long-term psychological syndromes that linger among medical professionals. Facing such facts, both policy makers and administrators need to ensure more support and assistance focused on frontline doctors. Their mental health can be improved or maintained primarily through two main bodies: the government and hospitals. The government should focus on providing certain seminars or training sessions for the counseling of their frontline doctors to secure and ensure their mental health. The hospitals and doctors' associations could regularly monitor their mental wellbeing and provide treatments to their HCW. Though effective strategies based on such research findings, Pakistan or other countries with similar conditions might be able to provide better medical conditions and staff support to effectively serve public health needs.

### 4.1. Limitations

Potential limitations associated with this study include its snowball, cross-sectional design, and inability to explore further effects. The cross-sectional research technique was incorporated as the only viable option under the pandemic conditions and constraints at that time, and the design proved unable to effectively examine the direct cause and effects among different factors. In this study, it was also a limitation that the samples gathered were mostly females (with no clear reasons why fewer male doctors responded), thus gender responses can be explored further, as well as what long-term effects might be noted regarding the mental health of either gender group. The responses are also noted to be time- and situation-sensitive and might change under future conditions, representing a common limitation of survey research. Fourth, the reliability of the participants' answers may be problematic because our study was conducted online. However, online data collection was not just preferable but the only viable option, as the survey was conducted during COVID-19 pandemic measures. Thus, conclusions drawn can only be tentative and generalized to situations like those examined. Future researchers could identify personality and situational factors that might directly be influencing the depression, anxiety, and stress symptoms of the doctors or compare the scores of DASS-21 with other developing or developed countries.

## 5. Conclusion

The mental wellbeing of the frontline doctors is a necessity for any country or nation during both normal and emergency situations like the COVID-19 pandemic. The current study has shed light on the alarming symptoms and higher-than-expected levels of depression, anxiety, and stress among the frontline doctors who have had to cope with the fifth wave of COVID-19 in Pakistan. Highlighting this issue warrants serious consideration from the government and both public and private hospitals' management. The policy makers in Pakistan or similar countries need to frame new polices to ensure their doctor's wellbeing which can ultimately influence the betterment of health in the society. The present study also validated the established DASS-21 instrument in the cultural context of Pakistan. Future research could seek to identify personality and situational factors that are directly influencing the depression, anxiety, and stress of doctors to consider correlations between factors or moderating variables. More studies targeting the long-term impacts of COVID-19 and the post-pandemic situation on the mental health of doctors or other health care populations will be beneficial for a greater understanding concerning the nature and influences of these past and future pandemics.

## Data availability statement

The raw data supporting the conclusions of this article will be made available by the authors, without undue reservation.

## Ethics statement

The studies involving human participants were reviewed and approved by the Institutional Ethics Review Committee of the SISU Intercultural Institute (SII), Shanghai International Studies University (SISU), China (2022-SII/IRB-0103). The patients/participants provided their written informed consent to participate in this study.

## Author contributions

MN: investigation, conceptualization, methodology, validation, data collection, formal analysis, and writing—original draft preparation. SK: conceptualization, methodology, resources, and writing—review and editing. IB: conceptualization, formal analysis, and writing—review and editing. All authors contributed to the article and approved the submitted version.
